# Hepatitis C virus cascade of care in the general population, in people with diabetes, and in substance use disorder patients

**DOI:** 10.1186/s13027-021-00345-8

**Published:** 2021-01-19

**Authors:** Olivera Djuric, Marco Massari, Marta Ottone, Giorgia Collini, Pamela Mancuso, Massimo Vicentini, Antonio Nicolaci, Angela Zannini, Alessandro Zerbini, Valeria Manicardi, Loreta A. Kondili, Paolo Giorgi Rossi

**Affiliations:** 1 Epidemiology Unit, Azienda Unità Sanitaria Locale-IRCCS di Reggio Emilia, Via Amendola 2, 42121 Reggio Emilia, Italy; 2 Center for Environmental, Nutritional and Genetic Epidemiology (CREAGEN), Section of Public Health, Department of Biomedical, Metabolic and Neural Sciences, University of Modena and Reggio Emilia, Modena, Italy; 3 Infectious Diseases Unit, Azienda Unità Sanitaria Locale–IRCCS di Reggio Emilia, Reggio Emilia, Italy; 4 Addiction Care Unit, Azienda Unità Sanitaria Locale–IRCCS di Reggio Emilia, Reggio Emilia, Italy; 5 Unit of Clinical Immunology, Allergy and Advanced Biotechnologies, Azienda Unità Sanitaria Locale–IRCCS di Reggio Emilia, Reggio Emilia, Italy; 6 Department of Internal Medicine, Hospital of Montecchio, Azienda Unità Sanitaria Locale-IRCCS di Reggio Emilia, Reggio Emilia, Italy; 7 National Center for Global Health, Istituto Superiore di Sanità, Rome, Italy

**Keywords:** Hepatitis C virus, Hepatocellular carcinoma, Cascade of care, Linkage to care, Addiction, Diabetes mellitus

## Abstract

**Background:**

The aim was to evaluate the hepatitis C virus (HCV) cascade of care in the general population (GP) and in two high-risk populations: patients with diabetes mellitus (DM) and substance users (AS) in treatment in Reggio Emilia Province, Italy.

**Methods:**

A population-based cross-sectional study was conducted that included 534,476 residents of the Reggio Emilia Province, of whom 32,800 were DM patients and 2726 AS patients. Age-adjusted prevalence was calculated using the direct method of adjustment based on the age-specific structure of EU population.

**Results:**

The prevalence of HCV testing was 11.5%, 13.8%, and 47.8% in GP, DM, and AS patients respectively, while HCV prevalence was 6.5/1000, 12.6/1000, and 167/1000, respectively. The prevalence of HCV RNA positivity was 4.4/1000, 8.7/1000, and 114/1000 in the three populations, respectively. The rates of HCV RNA-positive individuals not linked to care were 27.9%, 27.3%, and 26% in GP, DM, and AS patients, respectively, while the rates of those cured or cleared were 70.9%, 71%, and 69.9%, respectively. The prevalence of HCV testing was higher for females of reproductive age than for males the same age: 218.4/1000 vs. 74.0/1000, respectively. While more foreigners than Italians underwent the HCV test and were HCV positive, fewer foreigners than Italians received HCV treatment and were cured.

**Conclusions:**

The low HCV testing and linkage to care rates remain an important gap in the HCV cascade of care in Northern Italy. The prevalence of cured/cleared residents remains lower among foreigners than among Italians.

**Supplementary Information:**

The online version contains supplementary material available at 10.1186/s13027-021-00345-8.

## Introduction

Hepatitis C virus (HCV) affects approximately 180 million people globally, of whom 71 million have chronic hepatitis C virus infection [1]. Italy has the highest prevalence of HCV in Europe (up to 27.6%, depending on the region) [2]. Owing to the effectiveness of recently introduced pan-genotypic direct-acting antivirals (DAAs), The World Health Organization has established the global health sector strategy on eliminating viral hepatitis by 2030 [3]. Although Italy has developed a National Hepatitis Plan and is among those countries which are on track to achieving HCV elimination goals [4, 5], decentralized models of HCV care with ununiformed strategies across regional networks persist [6].

People who inject drugs (PWID) remain the largest reservoir of HCV in young and middle-aged adults in Italy and is the population with the highest prevalence of HCV [7]. Despite a documented decrease in HCV rates in this population thanks to harm reduction programmes, injecting drugs and iatrogenic transmission remain among the most common modes of HCV transmission and virus persistence in the population [8]. However, the proportion of PWID linked to adequate care remains low, which represents an important barrier to achieving the elimination goal set in Italy, despite the availability of DAAs and broad access to care [8].

After transplantation/recipience of substances of human origin and dialysis/haemodialysis, therapies and procedures related to the diabetes mellitus (DM) are the third most common iatrogenic mode of transmission of HCV [2]. There is increasing number of reports of an association between HCV and type-2 DM [9, 10], although their results concerning the direction and underlying mechanisms of this association are discordant. Nevertheless, numerous studies have observed a higher prevalence of HCV in the population with diabetes [11–24].

Similar to HCV prevalence, the availability of treatment and linkage to care also vary across Italy, and the actual number of HCV patients being cared for remains uncertain. Overall, the number of known chronically infected patients eligible for DAA treatment has decreased by half (from 308 thousand patients in 2015 to 160 thousand patients in 2019) [25]. Nevertheless, it has been estimated that there are still 300 thousand individuals unaware of their infection [26]. One simulation study estimated that a linkage to care of around 40–60% would lead to treating over 35,000 patients per year up to 2025. Such a strategy, without any kind of screening, would leave untreated a significant number of infected individuals who are unaware of their infection status [27]. Therefore, with the effective treatment available, the challenge of contemporary prevention and management of HCV is to identify and treat undiagnosed, at-risk, and hard-to-reach infected persons and to estimate compliance rates of the individual steps along the cascade of care. To achieve this, it is important to estimate the current percentage of HCV-positive people and those not yet linked to care and cured in order to provide important information necessary to eliminate HCV.

The primary objective of the study was to estimate the completeness of the HCV cascade of care steps in the general population of the Reggio Emilia Province and in two high-risk population groups: the population with diabetes and that of substance users treated in an addiction rehabilitation care service. The secondary objective was to determine the potential role of sociodemographic characteristics (sex, citizenship, and age) on the prevalence of cascade of care outcomes.

## Materials and methods

### Study design and population

A population-based cross-sectional study was conducted in which routinely collected data on HCV testing performed from July 1, 2008 to December 31, 2017 were analysed.

The general population consisted in all individuals resident in the Reggio Emilia Province on 31/12/2017 according to the resident population registry of the local health authority. The population with diabetes was all prevalent cases of diabetes on 31/12/2016 according to the provincial diabetes registry. Women with gestational diabetes or women receiving treatment for polycystic ovarian syndrome are excluded from the registry. The population of addiction rehabilitation service patients comprised all the patients who have been treated for mental or behavioural disorders due to the use of alcohol (ICD10 F10), opioids (ICD10 F11), cannabinoids (ICD10 F12), or cocaine (ICD10 F14) in the Addiction Service (AS) between January 2016 and March 2018 and who were resident of the Reggio Emilia Province in this period. Individuals attending smoking cessation programmes were excluded.

The study was approved by the Ethics Committee of Area Vasta Emilia Nord – IRST (4 December 2018).

### Endpoints and definitions

**Fig. 1:**
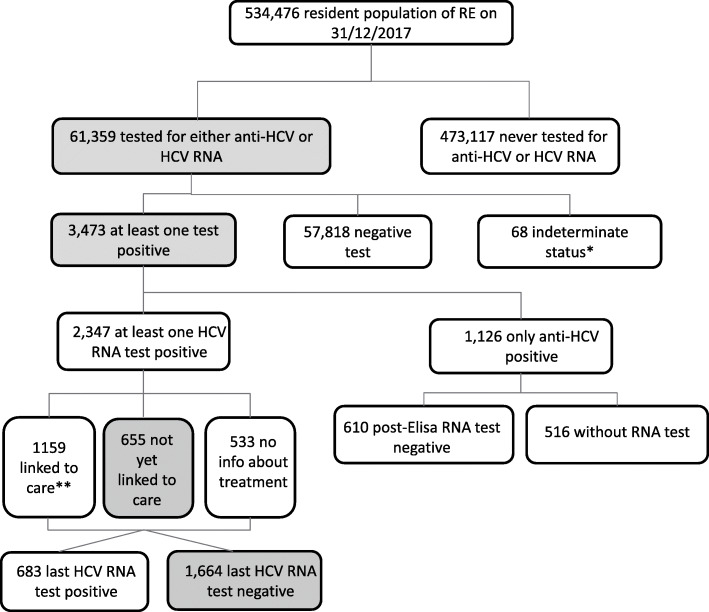
HCV cascade of care in the general population tested for HCV from 1/7/2008 to 31/12/2017. ^*^inconclusive result, haemolysed serum, blank tubes, reversed sample, sample not received, test not performed. ^**^Treatment data available for the period 2015–2019

**Fig. 2:**
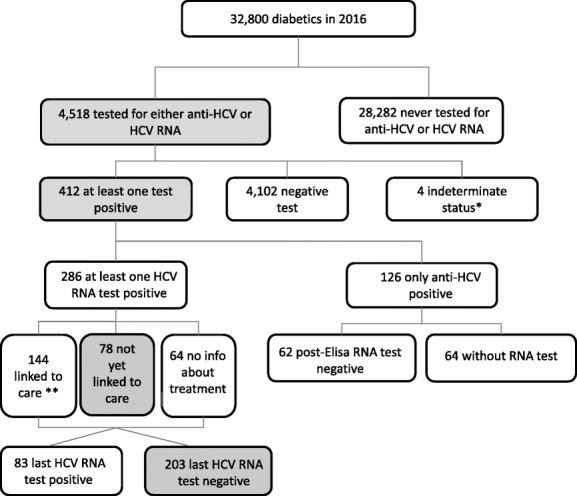
HCV cascade of care in the diabetic population tested for HCV from 1/7/2008 to 31/12/2017. ^*^inconclusive result, haemolysed serum, blank tubes, reversed sample, sample not received, test not performed. ^**^Treatment data available for the period 2015–2019

**Fig. 3:**
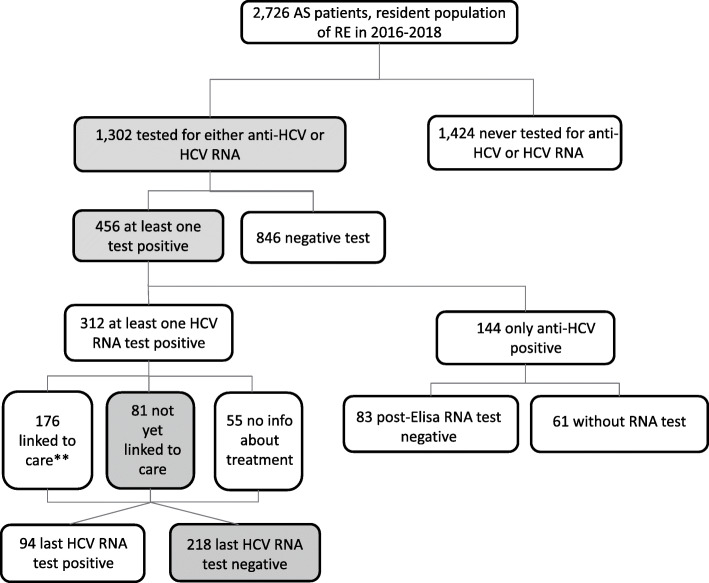
HCV cascade of care in the AS population tested for HCV from 1/7/2008 to 31/12/2017. ^*^inconclusive result, haemolysed serum, blank tubes, reversed sample, sample not received, test not performed. ^**^Treatment data available for the period 2015–2019

The endpoints of interest were: (a) prevalence of HCV testing (anti-HCV and/or HCV RNA); (b) prevalence of people with present infection markers or active infection (anti-HCV or HCV RNA positivity); (c) prevalence of people with known HCV RNA positivity who are still waiting for treatment; and (d) prevalence of those who were cured or cleared (patients whose last RNA test was negative among those who had a previous positive HCV RNA test) (Figs. 1, 2 and 3). The denominator for the first two outcomes was the entire population included in the study (general, diabetic, and AS clients), while the prevalence of last two outcomes (HCV RNA-positive persons who were still waiting for treatment and those who were cured/cleared) was calculated on the persons with at least one positive HCV RNA test during the study period. All outcomes were calculated for the general population and for the high-risk subgroup populations (people with diabetes and addiction rehabilitation service clients).

Subjects who had serological evidence of past or recent HCV infection, i.e., had at least one HCV test positive for presence of antibody (anti-HCV) or HCV RNA were considered HCV infected, while those who were HCV RNA-positive at least once during the study period were considered viraemic or actively infected at a certain moment in their life [28]. A second HCV RNA test was performed in 87.6% of positive HCV RNA tests (Supplementary table).

### Data sources and linkage

For the purpose of the study, data were retrieved from the resident population registry, the Reggio Emilia Diabetes Registry [29], the SistER (Sistema Informativo sulle Tossicodipendenze Regione Emilia Romagna) platform [30], the Reggio Emilia Local Health Authority (AUSL) laboratory information system [30], and the AUSL DAA treatment database (Supplementary figure).

The Reggio Emilia Diabetes Registry was created by deterministic linkage of six routinely used data sources: hospital discharge records, drug dispensation, biochemistry laboratory, disease-specific exemption, diabetes outpatient clinics, and mortality databases. Women with gestational diabetes or women receiving treatment for polycystic ovarian syndrome are excluded from the Diabetes Registry. The methods applied to develop this registry have been described in detail elsewhere [29].

The data for AS clients was extracted from SistER, a platform which serves as a database for the registration and follow up of patients with substance use disorders (SUD) in the Emilia-Romagna region who have been assisted by the AS. The AS registry includes all the people treated for addiction either in the Local Health Authority clinics or in private clinics that have an agreement with the Italian National Health Service. The AS carries out prevention, treatment, and rehabilitation of disorders due to addiction to legal and illegal psychoactive substances and to gambling [30].

The AUSL-IRCCS laboratory information system, a database containing laboratory results of all tests carried out in the Province’s public health network and coded using an internal classification, was used to estimate the prevalence of HCV-test-related outcomes [30].

Data on patients undergoing DAA treatment in the AUSL public outpatient clinics were available for the period 2015–2019.

### Statistical analysis

Outcomes are presented as absolute and relative frequencies. The crude and age-standardized prevalence rates were computed, with 95% confidence intervals, for each outcome in each of the three populations. Age-adjusted prevalence was calculated using the direct method of adjustment based on the age and sex-specific rates for the sample and age-specific structure of the EU population (EU standard population 2013). Prevalence ratios of HCV infection among tested patients were estimated in the three populations using Poisson regression models, adjusted for sex, age group, and citizenship. We used Stata 13.0 SE (Stata Corporation, Texas, TX) software package for the main analysis.

## Results

### Prevalence of outcomes related to HCV cascade of care

The presence of HCV infection was tested (either by HCV antibody or HCV RNA) in 61,359 residents over the nine-and-a-half-year period, with a coverage rate of 11.5%; the majority of residents (88.5%) were not tested (Fig. 1). Of those tested, 3473 (5.7%) had serologic evidence of infection (prevalence of people ever diagnosed with HCV infection markers or active infection 6.5/1000). There were 1126 residents who were only anti-HCV reactive, of whom 516 (45.8%) were not subsequently tested for the presence of HCV RNA. The prevalence of known ever-HCV RNA-positive people was 4.4/1000; of the 2347 ever actively infected residents, the last HCV RNA test of 1664 (70.9%) was negative either as a result of DAA treatment, other treatment, or as spontaneous clearance. The rate of people who were HCV RNA positive and not yet linked to care was 27.9%, while the probability of being linked to care, given the positive RNA test, was 49.4%.

Of the 32,800 registered diabetic patients, 4518 (13.8%) were tested for either HCV antibody or HCV RNA (Fig. 2). There were 412 (9.1%) diabetic patients with serologic evidence of infection (prevalence of people ever diagnosed with HCV infection markers or active infection: 12.6/1000) and 126 who were only anti-HCV reactive. Of these, 64 (50.8%) were not tested for HCV RNA. HCV RNA detection prevalence was 8.7/1000. The rate of actively infected diabetic patients not yet linked to care was 27.3%, while the probability of being linked to care, given the positive RNA test, was 50.3%. Of the 286 actively infected diabetic patients, the last HCV-RNA test of 203 (71%) was negative as a result of DAA treatment, other treatment, or as spontaneous clearance.

In the population of 2726 AS patients, the prevalence of those tested at least once for either HCV antibodies or HCV RNA was 47.8% (Fig. 3). Of those tested, 456 (35%) had serological evidence of infection (prevalence of people ever diagnosed with HCV infection markers or active infection 167.3/1000); 312 were only anti-HCV reactive. Of these, 61 (19.5%) did not undergo subsequent HCV RNA test. HCV RNA detection prevalence was 114.4/1000. The rate of actively infected AS patients not yet linked to care was 26% and the probability of being linked to care, given the positive RNA test, was 56.4%. Of the 312 actively infected AS patients, 218 (69.9%) were cured or cleared.

### Prevalence of outcomes related to HCV cascade of care by sex and citizenship

The prevalence of HCV testing was higher in females compared to males in all three populations (Table 1). Males in the general population and the diabetic population had in a higher rate of serologic evidence of HCV infection than did females [6.6 (95%CI 6.3–6.9) vs. 5.4 (95%CI 5.1–5.7) and 10.8 (95%CI 8.6–13.1) vs. 8.2 (95%CI 6.2–10.3), respectively)], while in the AS population females had in a higher rate of positivity to HCV infection than did males [123.8 (95%CI 96.4–151.2) vs. 113 (95%CI 99.8–126.2)].

**Table 1:** Crude and adjusted prevalence^a^ of HCV testing, positivity, cured or cleared, and last RNA positive waiting for treatment, according to sex

	General population		Diabetic population		AS patients	
	Crude (95%CI)	Adjusted (95%CI)	Crude (95%CI)	Adjusted (95%CI)	Crude (95%CI)	Adjusted (95%CI)
**HCV tested**						
Female	148.7 (147.2–150.1)	152.4 (151.1–153.7)	143.8 (137.8–150.1)	179.1 (164.7–193.6)	555.6 (490.1–626.6)	451.4 (390.3–512.5)
Male	79.8 (79.7–80.9)	78.4 (77.3–79.4)	132.7 (127.4–138.2)	122.9 (109.0–136.8)	461.1 (433.5–490.0)	367.8 (336.9–398.8)
**HCV positive**						
Female	6.0 (5.7–6.3)	5.4 (5.1–5.7)	12.5 (10.7–14.4)	8.2 (6.2–10.3)	184.5 (148.0–227.3)	123.8 (96.4–151.2)
Male	7.0 (6.7–7.3)	6.6 (6.3–6.9)	12.6 (11.0–14.4)	10.8 (8.6–13.1)	163.6 (147.3–181.2)	113.0 (99.8–126.2)
**Cured or cleared**						
Female	677.5 (629.3–728.4)	692.2 (644.5–739.9)	644.1 (507.5–806.1)	337.1	571.4 (390.9–807.7)	458.4
Male	735.8 (689.3–784.6)	746.2 (716.1–776.3)	756.0 (630.2–899.4)	526.4	726.7 (625.9–838.8)	432.5
**Last RNA+ waiting for treatment**						
Female	317.0 (284.3–352.4)	304.0 (256.3–351.6)	347.5 (249.3–471.4)	191.1	392.9 (246.2–594.8)	176.2
Male	246.8 (220.0–275.8)	234.5 (204.8–264.1)	220.2 (155.1–303.6)	121.2	230.5 (175.4–297.3)	295.3

*AS* Addiction Service

^a^Per 1.000

While foreigners in the general population had a higher rate of being tested for HCV and a higher rate of serological evidence of HCV infection, a lower percentage of them was linked to care and cured compared to Italian residents (Table 2). In the diabetic population, foreign residents had a higher rate of being tested for HCV than did Italian residents, but the prevalence of having serological evidence of HCV infection [9.5 (6.4–12.7) vs. 10.2 (8.2–12.2)] and linkage to care were similar in both (128.1 vs. 130.6). All these outcomes in AS patients (except for HCV RNA-positives waiting for treatment) were more prevalent in Italian compared to foreign residents.

**Table 2:** Crude and adjusted prevalence^a^ of HCV testing, ever positivity, cured or cleared, and RNA positive not yet treated, according to citizenship

	General population		Diabetic population		AS patients	
	Crude (95%CI)	Adjusted (95%CI)	Crude (95%CI)	Adjusted (95%CI)	Crude (95%CI)	Adjusted (95%CI)
**HCV tested**						
Foreign	145.2 (142.4–148.1)	122.4 (119.6–125.2)	158.2 (143.0–174.5)	160.7 (140.0–181.3)	525.8 (448.2–613.0)	325.5 (280.1–371.0)
Italian	110.3 (109.3–111.2)	111.7 (110.8–112.6)	136.0 (131.9–140.3)	142.4 (130.8–154.1)	484.5 (456.7–513.4)	388.0 (359.5–416.4)
**HCV positive**						
Foreign	6.2 (5.6–6.8)	7.3 (6.2–8.3)	15.8 (11.3–21.5)	9.5 (6.4–12.7)	100.0 (67.9–141.9)	71.3 (42.1–100.5)
Italian	6.5 (6.3–6.8)	5.8 (5.6–6.0)	12.3 (11.1–13.6)	10.2 (8.2–12.2)	180.8 (164.0–198.8)	121.5 (109.0–133.9)
**Cured or cleared**						
Foreign	680.3 (580.8–792.1)	597.3	720.0 (426.7–1137.9)	486.9	571.4 (246.7–1125.9)	305.0 (216.6–393.4)
Italian	712.3 (677.7–749.3)	675.6 (630.4–720.8)	708.8 (610.3–818.6)	518.2	704.7 (612.6–806.7)	434.3
**Last RNA+ not yet treated**						
Foreign	311.5 (245.4–389.9)	272.6	280.0 (112.6–576.9)	128.1	428.6 (157.3–932.8)	335.0
Italian	275.3 (253.3–298.7)	308.8 (264.0–353.7)	272.0 (212.5–343.1)	130.6	251.7 (198.0–315.5)	184.6

*AS* Addiction Service

^a^Per 1.000

**Fig. 4:**
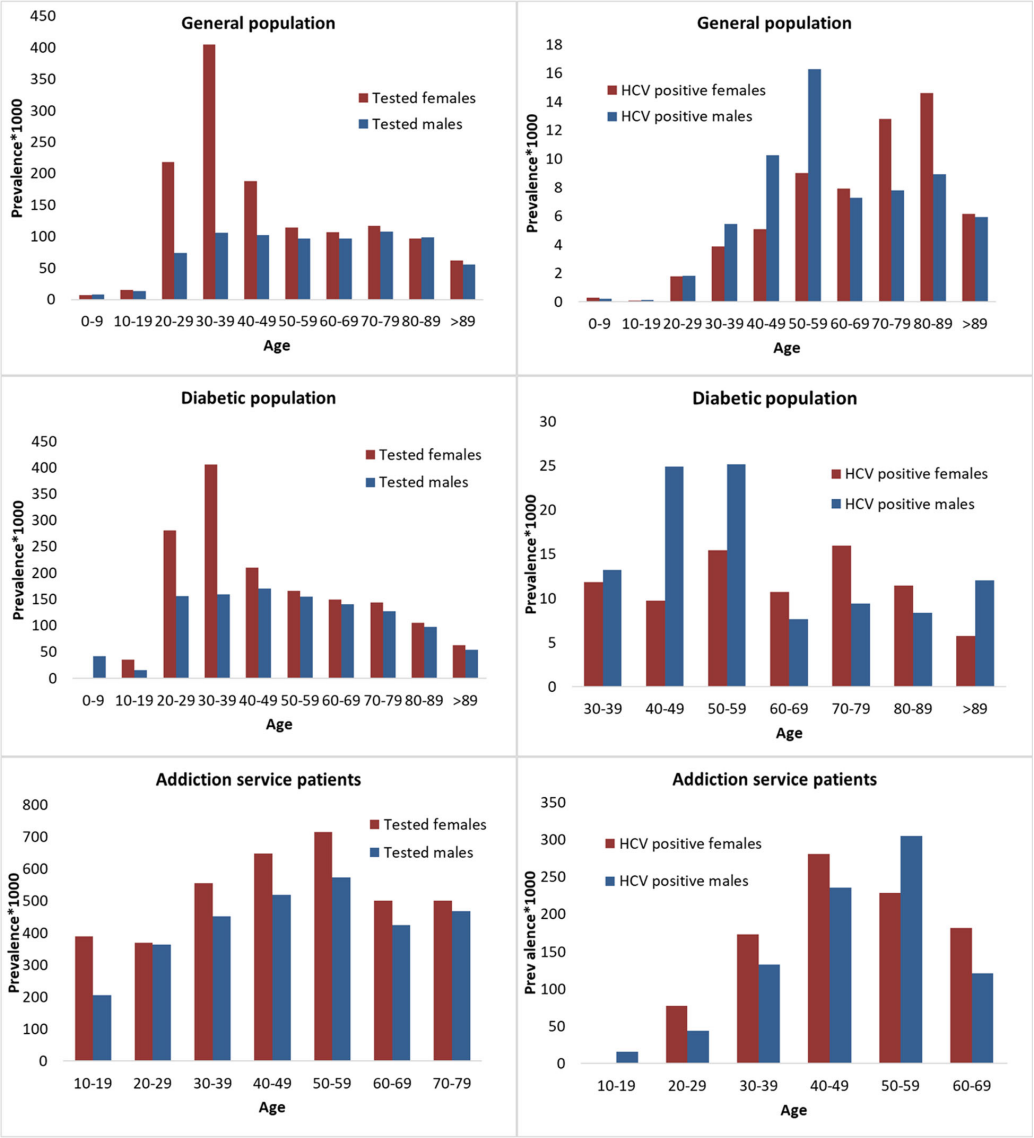
Prevalence of HCV tested and HCV-positive individuals by age and sex

**Fig. 5:**
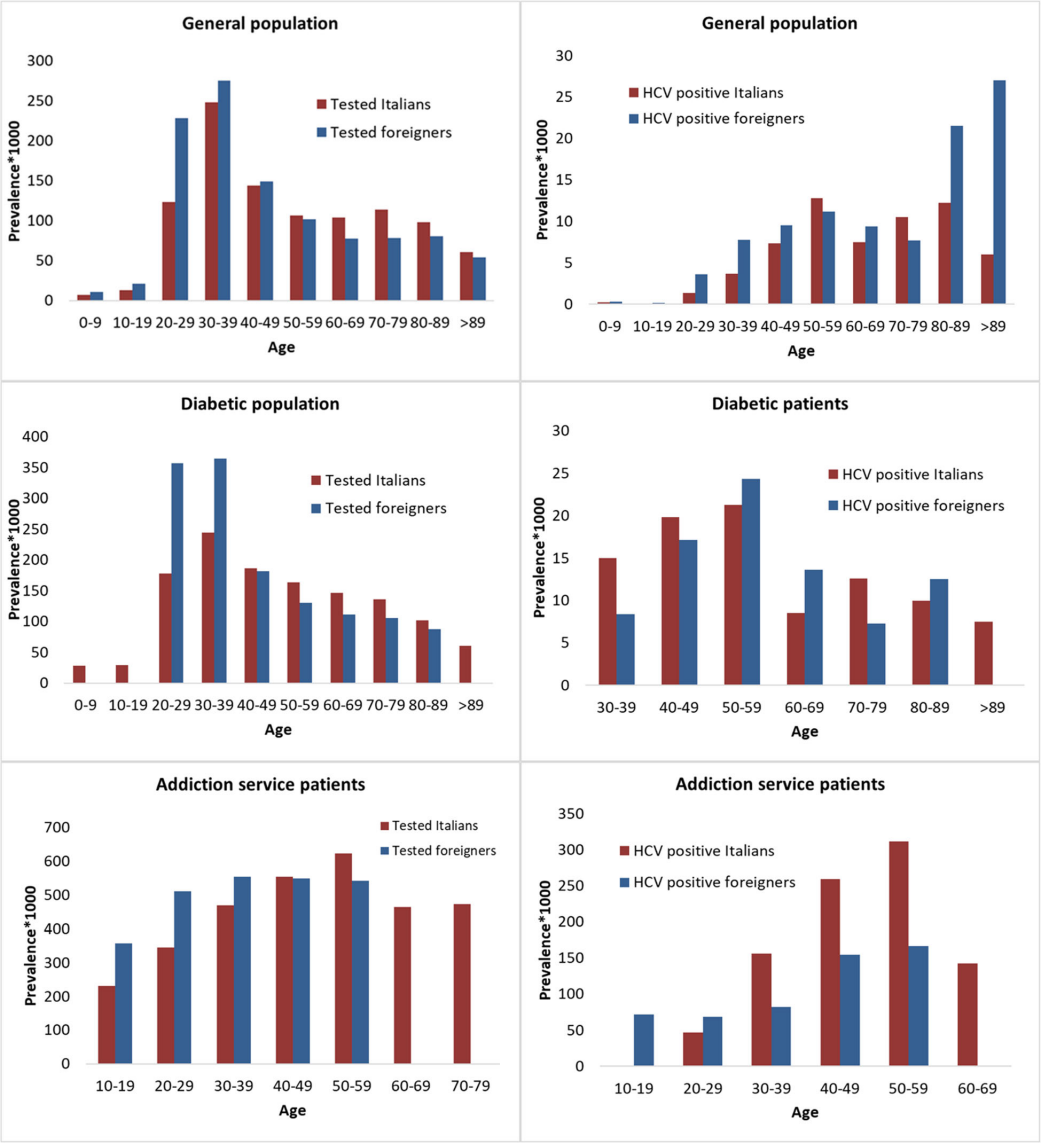
Prevalence of HCV tested and HCV-positive individuals by age and citizenship

The prevalence of HCV testing was higher for females of reproductive age than for males the same age: 218.4/1000 vs. 74.0/1000, respectively, for age group 20–29 years; 404.5/1000 vs. 106.4/1000, respectively, for age group 30–39 years; and 187.6/1000 vs. 102.9/1000, respectively, for age group 40–49 years (Fig. 4). The highest HCV prevalence for men was observed in those aged 50–59 years (Fig. 5). For the diabetic population and for AS patients, an increase in prevalence was seen up to the age group of 50–59 years, when it peaked and then started to decline.

### Prevalence of people ever diagnosed with HCV infection markers or active infection (anti-HCV or HCV RNA) by sex and citizenship

In the general population, the prevalence of ever-positive tests among females was two times lower than among males (prevalence ratio [PR] 0.49, 95%CI 0.46–0.53), while people over age 60 years had almost double the prevalence of those younger than 60 years (PR 1.84, 95%CI 1.72–1.98), when adjusted for other characteristics in the model (Table 3). In the diabetic population only, age was associated with prevalence of ever-positive tests (PR for people over age 60 years was 0.75, 95%CI 0.61–0.93). In the population of AS patients, the PR for those over age 60 years was 0.66 (95%CI 0.37–1.18) and PR for foreigners was 0.50 (95%CI 0.35–0.72).

**Table 3:** Prevalence ratios with 95% confidence intervals for HCV RNA detection, adjusted for sex, age, and citizenship

	General population		Diabetic population		AS patients	
	PR	95%CI	PR	95%CI	PR	95%CI
Sex						
Male	Ref.		Ref.		Ref.	
Female	0.49	0.46–0.53	0.92	0.76–1.12	0.93	0.73–1.16
Age						
< 60 years	Ref.		Ref.		Ref.	
≥ 60 years	1.84	1.72–1.98	0.75	0.61–0.93	0.66	0.37–1.18
Citizenship						
Italian	Ref.		Ref.		Ref.	
Foreign	0.95	0.86–1.06	0.98	0.69–1.38	0.50	0.35–0.72

## Discussion

A reliable estimation of the prevalence of HCV and other principal steps in the cascade of care in the general population and in key high-incidence groups is fundamental to understanding the current epidemiological landscape and to developing action plans and suitable scale-up interventions to facilitate the elimination of HCV.

In line with the literature, HCV test coverage and prevalence of anti-HCV reactivity and HCV RNA detection in our study were highest in AS patients and lowest in the general population. Interestingly, the overall prevalence of those who were anti-HCV positive and had not yet undergone the RNA test and of those who were currently infected (HCV RNA-detected) but not yet linked to care were only slightly lower in the population of AS patients. As for viraemic subjects in the general population, diabetic population, and AS patients, the probability of being linked to care was similar (49.4%, 50.3%, and 56.4%, respectively), suggesting that although there is a substantial number of patients who still need to be tested for RNA and linked to care, the availability of HCV testing and linkage to care for hard-to-reach populations (diabetic patients and people with SUD) are similar to that of the general population.

The direction of the association between diabetes and HCV remains debated. However, most studies report a higher prevalence of HCV in patients with DM than in controls [11–24], while a few found no difference [31–33]. Nevertheless, the prevalence of people in our study with evidence of HCV infection was almost two times higher in the diabetic population than in the general population, despite the similar testing rate.

With the limitation of jointly calculating outcomes for individuals with alcohol or drug use addiction disorder in our study, test uptake, serological evidence of HCV infection markers (anti-HCV and/or HCV RNA positive), and active HCV infection (HCV RNA detected) among AS patients were almost identical to those reported by Øvrehus et al. in a single large register-based study conducted on Danish people in treatment for drug addiction [34]. However, they were notably lower than those previously reported in Italy [35, 36]. This is consistent with the decrease in overall HCV prevalence in Italy observed in the last 5 years and could be partly explained by the more-than-double testing rate and linkage-to-care rate found in our study and in the Emilia-Romagna region compared to what has been previously reported in Italy as a whole [35]. Nevertheless, this is likely an underestimate of the real HCV prevalence in the population with substance dependence since not all substance users have the same risk of HCV infection, and those who are at higher risk of HCV (persons who inject drugs-PWID and those who receive oral opioid substitution therapy-OST) are those who, despite the higher uptake of HCV test [37], are not reached by addiction treatment centres or who have a high interruption rate of OST [37, 38]. In Reggio Emilia, marginalized individuals, in particular those who inject drugs and have present or past history of homelessness, are reached by the Harm Reduction Program put in place by AS, which provides them with sterile syringes (Needle and Syringe Program), opioid substitution therapy, and alcohol detoxification treatment. AS professionals also actively propose HCV testing, but compliance with testing is low in this population.

Similar to drug addiction, and despite insufficient data related to the impact of past or recent alcohol use on outcomes with DAA therapy, one study showed that recent alcohol use resulted in a higher treatment discontinuation rate and therefore a lower sustained virologic response (SVR) achievement rate [39]. Point-of-care single-step testing for HCV infection, such as reflex HCV testing, could decrease the number of medical appointments currently necessary in the multistep testing protocol and which has been identified as main obstacle to HCV diagnosis and treatment [40]. This could be particularly beneficial to at-risk groups, such as diabetic patients, those undergoing haemodialysis, or those attending an addiction treatment centre, who already have to deal with multiple medical appointments over the course of their care.

The prevalence of cure or spontaneous clearance, estimated as the percentage of previously HCV RNA-reactive patients linked to care whose posttreatment HCV RNA test was negative, is one of the key indicators of cascade of care efficiency in the HCV care continuum and greatly depends on the linkage to care rate. In our study, the percentage of HCV-reactive people not yet linked to care was substantially lower than the overall European rate in 2017 as well as that in the USA, where only 9.3% of chronic HCV-infected people successfully completed all steps of the cascade of care all the way to achieving sustained virologic response [41, 42]. Despite the relatively high overall linkage to care in our study, the percentage of cured/cleared foreigners was lower for than that of Italians; the former, despite being more often tested, had lower access to treatment. Besides biological differences, this can be explained by barriers to accessing care that foreigners encounter compared to Italians and by the higher antiviral therapy discontinuation rate in this group [43]. Despite therapy being completely free for regular immigrants, accessibility to health services is not merely an issue of insurance coverage. There could be problems related to language or integration in our society that make it difficult to navigate the bureaucracy [44], and there could also be issues of distrust in public institution because, while in this paper we included only resident immigrants, some of them may have been waiting for the renewal of their residence permit.

In our study, the highest HCV prevalence for men was observed in those aged 50–59 years. For the diabetic population and for AS patients, an increase in prevalence was seen up to age 50–59 years, when it peaked and then started to decline. Such an age distribution among elderly males in the general population and middle-aged diabetic patients and AS patients is in line with the HCV age dynamic previously reported in Italy and may be a reflection of the two big epidemic waves of HCV observed in the last century [45, 46]. The first wave was due to unsafe health care procedures and the widespread use of outpatient invasive medical procedures and of medicines administered by parenteral injections with multi-use syringes in the years after World War II (the 1950s and ‘60s) [47]. The second wave occurred in the following two decades (1980–1990 as reported by Andreoni et al) and was due to increasing number of intravenous drug users [48].

We acknowledge several limitations of our study. In the absence of data on the achievement of a sustained virologic response, we calculated the percentage of the last not-reactive RNA test after the initial reactive one as a proxy for cure/clearance prevalence. As regards the high-risk populations, the generalisability of results to all alcohol and drug users is limited since the people dependent on substances other than opioids are underrepresented in our study. Even though cannabinoids and cocaine are the most abused substances in Italy, users are not usually referred to an addiction treatment centre. Up to 90% of alcohol-dependent people and more than 60% of high-risk opioid users in Italy do not seek or have not been referred to rehabilitation or drug replacement treatment [49], and it has been estimated that more than half of the HCV-infected population are former PWIDs who have permanently stopped injecting drugs and might belong to the hard-to-reach population if they are not treated in the AS clinic [50].

The strength of this study is that it is the largest observational study which unifies and compares all important HCV infection- and treatment-related outcomes of HCV cascade of care, across the general population and in two high-risk groups (diabetic patients and addiction treatment centre patients) in a province in Northern Italy. By conducting a population-based study that includes all the residents of the Reggio Emilia Province, the limitation of previous studies, which did not include prisoners, men having sex with men, people in household contact with an HCV-positive subject, and other high-risk populations, was overcome. This population-based study also included homeless individuals, since the Italian National Health Service provides free-of-charge medical assistance to all Italian residents, including the unemployed and the homeless, who represent populations massively affected by HCV infection but who were not considered in previous studies.

## Conclusions

HCV testing remains a major gap in HCV cascade of care in Northern Italy; the majority of residents as well as two high-risk populations (diabetic patients and people in treatment for substance abuse) of one province have never been tested for HCV infection. In addition, the vast majority of those tested for HCV infection have never undergone a HCV RNA test, meaning that the proportion of actively infected people eligible for treatment is underestimated.

To achieve the goal of an HCV infection-free population, continued efforts are needed to reduce the gaps in HCV cascade of care in Italy. Efficient scaled-up interventions must be developed to capture hard-to-reach or disadvantaged populations, and screening strategies among those already in medical care must be optimized to earlier testing and linkage to care to reduce HCV prevalence and the further transmission of the virus.

## Supplementary Information

**Additional file 1: Supplementary figure**. Flowchart of data linkage and extraction.

**Additional file 2: Supplementary table**. Descriptive table on 2347 patients having at least one HCV RNA test positive: the detail of the number of repeated tests (starting from the first positive HCV RNA test).

## Data Availability

The datasets used and/or analysed during the current study are available from the corresponding author on reasonable request.

## Abbreviations

SUD: Substance use disorder
SVR: Sustained virologic response
HCV: Hepatitis C virus
AS: Addiction Service
DM: Diabetes Mellitus
GP: General population
RNA: Ribonucleic acid
DAA: Direct-acting antivirals
PWID: People who inject drugs
