# Supplementary figures and images for: Hepatitis C virus cascade of care in the general population, in people with diabetes, and in substance use disorder patients
Source: Infect Agent Cancer. 2021 Jan 19;16:5. doi: 10.1186/s13027-021-00345-8 (PMC7816419; doi:10.1186/s13027-021-00345-8)

**Supplementary figure**.


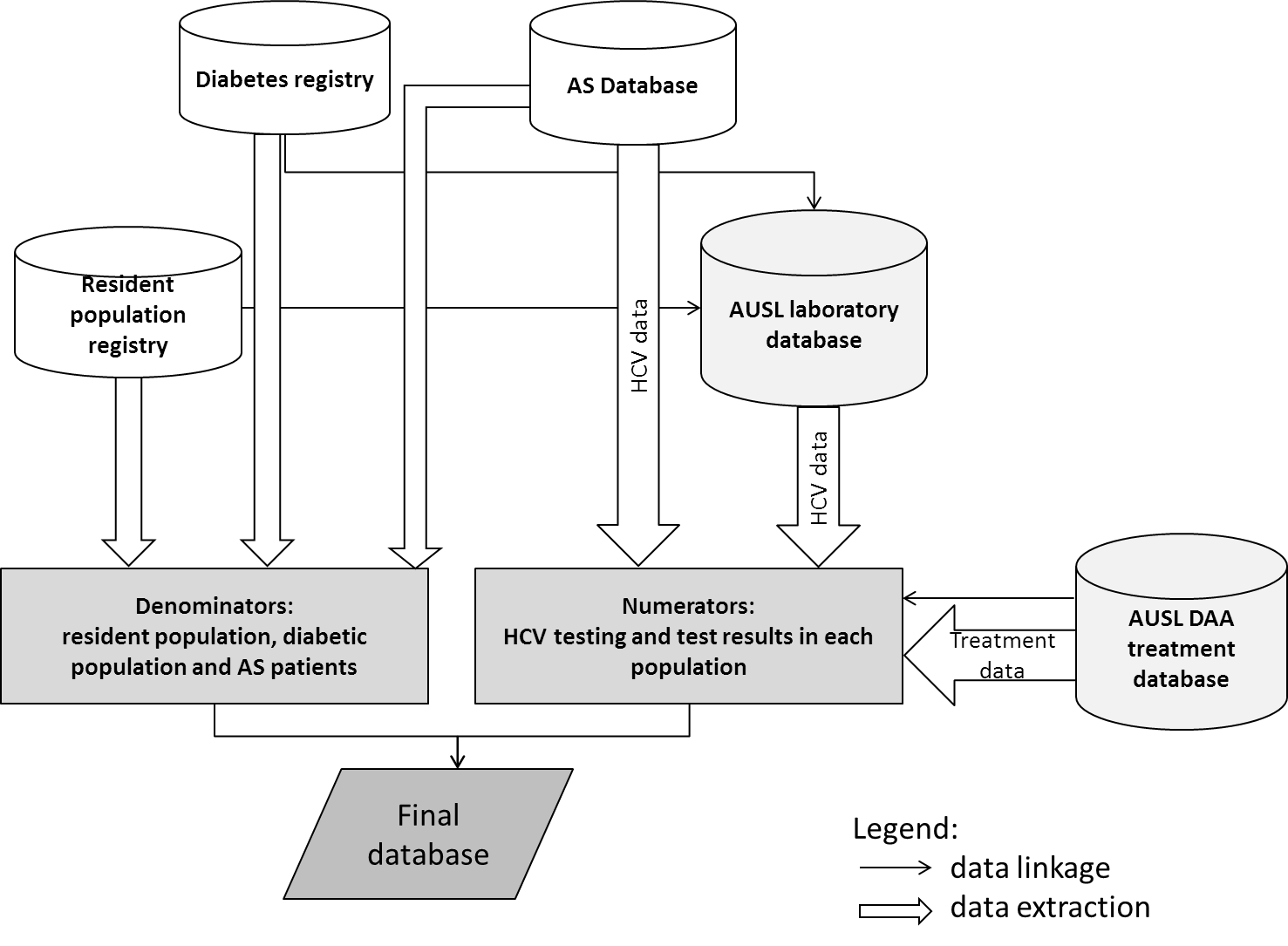

Supplement: Supplementary file 1 — Additional file 1: Supplementary figure. Flowchart of data linkage and extraction. [file 13027_2021_345_MOESM1_ESM.doc]
