# Supplementary material for: Hepatitis C virus cascade of care in the general population, in people with diabetes, and in substance use disorder patients
Source: Infect Agent Cancer. 2021 Jan 19;16:5. doi: 10.1186/s13027-021-00345-8 (PMC7816419; doi:10.1186/s13027-021-00345-8)
Supplement: Supplementary file 2 — Additional file 2: Supplementary table. Descriptive table on 2347 patients having at least one HCV RNA test positive: the detail of the number of repeated tests (starting from the first positive HCV RNA test). [file 13027_2021_345_MOESM2_ESM.docx]

*Supplementary table.* Descriptive table on 2,347 patients having at least one HCV RNA test positive: the detail of the number of repeated tests (starting from the first positive HCV RNA test).

|  | **General population** |
| --- | --- |
|  | **N** |
| HCV-RNA positive cases | 2,347 |
| No other test | 290 |
| Consecutive HCV-RNA test done | 2057 |
| 2 or more test positive | 360 |
| 1 or more test positive and 1 test negative | 1,544 |
| Alternation of positive and negative, and last positive | 33 |
| Alternation of positive and negative, and last negative | 120 |
